# Intrapartum Molecular Detection of Group B *Streptococcus*: Real-World Evaluation of Multiple Point-of-Care Platforms and the Potential Role of Lysis Efficiency

**DOI:** 10.3390/microorganisms14051060

**Published:** 2026-05-08

**Authors:** Mehdi Serrari, Lorenza Bianchi, Marie Tré-Hardy, Sara Törnblom-Paulander, Manon Alexandre, Arnaud Nevraumont, Ingrid Beukinga, Frédéric Buxant, Hamza Bensaoud, Laurent Blairon

**Affiliations:** 1 Department of Laboratory Medicine, Iris Hospitals South, 1050 Brussels, Belgium; 2 Department of Obstetrics and Gynaecology, Iris Hospitals South, 1050 Brussels, Belgium; 3 Faculty of Medicine, Université Libre de Bruxelles, 1070 Brussels, Belgium; 4 Department of Pharmacy, Namur Research Institute for Life Sciences, University of Namur, 5000 Namur, Belgium

**Keywords:** group B *Streptococcus*, intrapartum screening, point-of-care testing, polymerase chain reaction, loop-mediated isothermal amplification, molecular diagnostics, colonisation dynamics, real-life conditions

## Abstract

Antenatal screening for Group B Streptococcus (GBS) does not always reflect intrapartum colonisation status, and rapid molecular point-of-care tests (POCT) have been developed to enable real-time detection during labour. This prospective single-centre study evaluated the performance of six molecular assays (easyNat, FlashDetect, GenDx, GenPad, iPonatic, Revogene) and one antigen-based test (TZcheck) for intrapartum GBS detection under real-world conditions. Vaginal–rectal swabs were collected at admission from 104 women at ≥ 37 weeks of gestation and tested directly without prior enrichment, using conventional intrapartum culture as the reference standard. Diagnostic performance varied substantially across platforms, with positive percent agreement ranging from 0.0% to 80.6%, while negative percent agreement was generally high, except for GenDx. Seven culture-positive samples yielded negative results across all molecular assays, while one sample was consistently positive across multiple molecular platforms despite negative culture. Exploratory observations suggest that differences in lysis procedures may contribute to variability in assay performance, although this could not be formally assessed. These findings highlight the variability of intrapartum molecular POCT under routine conditions and underscore the need for cautious clinical interpretation and local validation prior to implementation.

## 1. Introduction

Group B *Streptococcus* (*Streptococcus agalactiae*, GBS) remains a leading cause of neonatal early-onset sepsis, pneumonia, and meningitis worldwide. Prevention strategies in many countries rely on universal antenatal screening of pregnant women between 35 and 37 weeks of gestation using vaginal–rectal culture. Women identified as carriers are offered intrapartum antibiotic prophylaxis (IAP), a strategy that has substantially reduced the incidence of neonatal early-onset GBS disease over the past decades [1,2,3].

Despite its effectiveness, antenatal screening provides only a temporal estimate of maternal colonisation status. GBS carriage is dynamic, and colonisation detected several weeks before delivery may not accurately reflect intrapartum status. Previous studies have shown that some women initially testing negative may become colonised before delivery, whereas others initially positive may no longer carry the organism at term [4]. Because routine screening is performed weeks before labour, these changes often remain undetected in clinical practice, potentially leading to both unnecessary antibiotic exposure and missed opportunities for prophylaxis.

Performing microbiological testing at the time of admission to the delivery unit would theoretically provide a more accurate assessment of colonisation status. However, conventional culture-based methods are not compatible with intrapartum decision-making. Standard laboratory workflows rely on selective enrichment in LIM broth followed by subculture on differential media such as Granada agar, requiring at least 24 h of incubation and additional reading at 48 h. Consequently, results are not available during labour and cannot guide the timely administration of intrapartum prophylaxis [5].

Rapid nucleic acid amplification tests (NAATs) have therefore been developed to provide near-patient detection of GBS directly from vaginal–rectal swabs during labour. Among these assays, the Xpert^®^ GBS system (Cepheid, Sunnyvale, CA, USA) has been the most extensively evaluated and has demonstrated high diagnostic performance in several studies [6]. Other platforms, including the Revogene^®^ GBS assay (Meridian Bioscience, Cincinnati, OH, USA), have also shown favourable results in intrapartum settings [7,8].

However, reported diagnostic performances vary substantially across studies and may depend on study design and pre-analytical conditions. Some evaluations have been conducted under controlled laboratory conditions or have incorporated enrichment steps prior to amplification. While such approaches may improve analytical sensitivity, they reduce the operational advantage of rapid testing in the delivery room, where results must be available within a short timeframe. In addition, real-world intrapartum samples may contain blood, mucus, or amniotic fluid, and may be collected in the context of ruptured membranes, all of which can affect assay performance and are not always represented in controlled validation studies.

Beyond these clinical and logistical considerations, biological factors may also play a role. The thick peptidoglycan-rich cell wall of Gram-positive bacteria such as GBS may limit nucleic acid release, particularly in direct testing conditions without enrichment, thereby affecting the sensitivity of molecular assays depending on the efficiency of lysis and extraction procedures.

In this context, pragmatic evaluations performed under routine delivery-unit conditions are essential to better understand the clinical performance of rapid intrapartum GBS assays. The present prospective study therefore evaluated several rapid molecular platforms and one antigen-based assay for intrapartum GBS detection under real-world conditions, using direct testing from vaginal–rectal swabs collected at admission to the delivery ward without prior enrichment, and compared their performance with conventional culture.

## 2. Materials and Methods

### 2.1. Study Design and Population

This prospective, single-centre study was conducted in the delivery unit of a 500-bed public general hospital in the Brussels-Capital Region, Belgium, between 20 June 2025 and 15 December 2025. All women admitted for labour at ≥37 weeks of gestation during the study period were eligible. All eligible women were consecutively invited to participate, provided that routine antenatal screening for GBS had been performed at 35—37 weeks of gestation and that written informed consent was obtained. 

Women were excluded if they had documented GBS bacteriuria during the current pregnancy, had received systemic antibiotic therapy within the 72 h preceding admission (to minimise potential culture suppression and molecular-culture discordance related to recent antimicrobial exposure), or declined participation.

The study protocol was approved by the local Ethics Committee (approval number: CE HIS 2025/04, approved on 18 February 2025) and conducted in accordance with the Declaration of Helsinki.

### 2.2. Sampling Procedure

At admission, vaginal–rectal specimens were collected by the attending obstetrician during routine clinical examination. Five separate swabs were obtained sequentially for parallel testing.

Swabbing followed standard antenatal screening procedures (lower vaginal followed by rectal sampling using the same swab). The order of swab collection was not standardised and varied between patients, reflecting real-world clinical practice and avoiding artificial standardisation.

All rapid assays were performed by trained laboratory personnel in close proximity to the delivery ward, ensuring immediate processing of samples after collection. This approach preserved intrapartum testing conditions while allowing strict adherence to manufacturer procedures.

### 2.3. Conventional Culture

For conventional microbiological detection of GBS, one vaginal–rectal swab was inoculated into LIM selective enrichment broth (Todd-Hewitt broth supplemented with colistin and nalidixic acid; Becton Dickinson, Sparks, MD, USA) and incubated for 18–24 h at 35 ± 1 °C under aerobic conditions.

Following enrichment, subculture was performed onto Granada agar plates (Becton Dickinson, Sparks, MD, USA). Plates were incubated in anaerobic conditions using an Anoxomat™ system (Advanced Instruments Inc., Norwood, MA, USA) and examined after 24 h. Plates were re-incubated under anaerobic conditions and re-examined at 48 h if negative at first reading. No direct plating was performed prior to enrichment.

Presumptive GBS colonies were identified by MALDI-TOF mass spectrometry using the Bruker Biotyper system (Bruker Daltonics GmbH, Bremen, Germany) according to the manufacturer’s instructions.

Conventional culture was used as the reference standard for performance evaluation, despite its known limitations in sensitivity.

### 2.4. Rapid Molecular and Immunochromatographic Assays

In addition to conventional culture, six rapid NAATs and one antigen-based immunochromatographic test (ICT) (TZcheck, Tongzhou Biotechnology Co., Ltd., Hangzhou, Zhejiang, China) were evaluated as a non-molecular comparator.

All assays were performed intrapartum directly from vaginal–rectal swabs, without prior enrichment, and strictly according to the manufacturers’ instructions. No protocol modifications were introduced, except where mechanical lysis was required as part of the recommended procedure. Operators were blinded to culture results at the time of testing.

The evaluated molecular platforms comprised real-time PCR-based assays as well as loop-mediated isothermal amplification (LAMP) assays, while the ICT relied on antigen detection. Reported turnaround times ranged from approximately 15 min for the ICT assay to 22—78 min for molecular assays.

All molecular tests were performed in parallel using dedicated swabs collected at admission. In order to preserve intrapartum applicability and real-time decision-making, no enrichment step was performed prior to molecular testing.

When specified by the manufacturer, mechanical lysis by sonication was performed as described below.

The evaluated platforms and their main technical characteristics, including methodological principles, turnaround time, and pre-analytical processing requirements, are summarised in Table 1.

### 2.5. Nucleic Acid Extraction and Lysis Procedures

Nucleic acid extraction procedures varied between platforms. Depending on the system, extraction and amplification were either fully integrated within a closed cartridge-based device or required an external lysis step prior to amplification.

For several platforms, nucleic acid extraction was performed automatically within the analytical system according to the manufacturer’s instructions. For others, an external lysis step was required before amplification.

For the FlashDetect assay (Coyote Bioscience Co., Ltd., Beijing, China), mechanical lysis by external sonication was mandatory for GBS detection and was performed using the manufacturer-compatible Coyote sonicator. Sonication was carried out for 90 s prior to cartridge loading, strictly following the manufacturer’s instructions. No additional enzymatic or chemical pre-treatment was applied beyond the procedures specified by the respective manufacturers. No additional optimisation was performed beyond manufacturer instructions.

### 2.6. Statistical Analysis

Conventional intrapartum culture was used as the reference standard for agreement analyses. Diagnostic performance of each rapid assay was assessed using positive percent agreement (PPA) and negative percent agreement (NPA) relative to culture results. Positive predictive value (PPV) and negative predictive value (NPV) were also calculated. All performance estimates were reported with two-sided 95% confidence intervals.

Agreement was interpreted according to standard thresholds for Cohen’s kappa coefficient (κ < 0.20: slight; 0.21–0.40: fair; 0.41–0.60: moderate; 0.61–0.80: substantial; >0.80: almost perfect agreement). Discordant results were further analysed by examining concordance across molecular assays. Cases with consistent positive results across multiple molecular platforms despite negative culture were considered suggestive of potential culture false-negative results, although no formal composite reference standard was predefined. This exploratory analysis was not used to recalculate primary performance metrics.

Statistical analyses were performed using MedCalc software Version 23.5.2 (MedCalc Software Ltd., Ostend, Belgium).

## 3. Results

**Figure 1:**
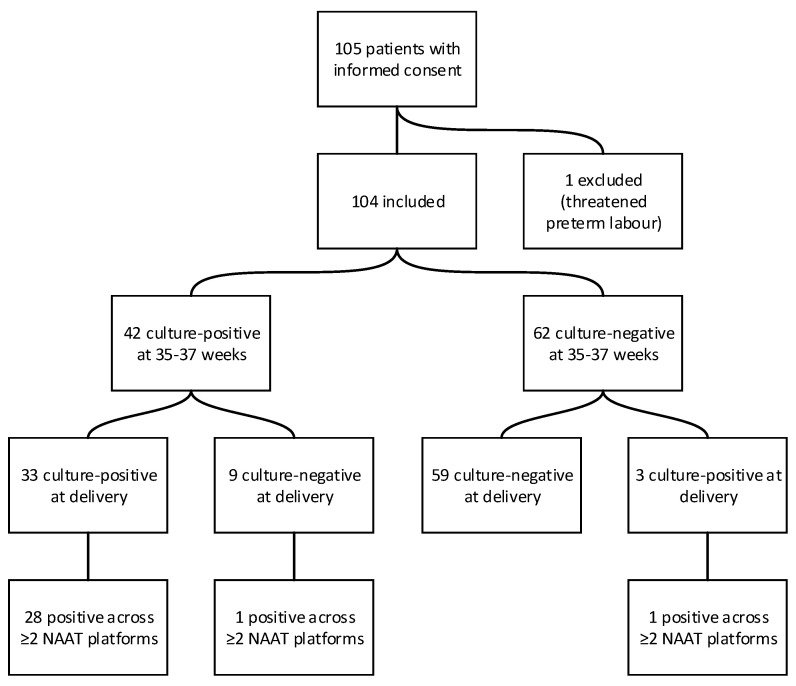
Flow diagram of study population and concordance between antenatal screening, intrapartum culture, and molecular testing.

A total of 104 women were included in the study. Based on antenatal screening at 35–37 weeks of gestation, 42 women were classified as GBS-positive and 62 as GBS-negative. The mean age was 31.7 years (range: 19–47). Fifteen women had a documented history of GBS colonisation during a previous pregnancy. Patient inclusion and changes in colonisation status between antenatal screening and delivery are shown in Figure 1.

At delivery, changes in colonisation status were observed in 12 cases: 9 of 42 women initially classified as positive (21.4%) became culture-negative, while 3 of 62 initially negative women (4.8%) were found to be culture-positive.

Diagnostic performance varied markedly across platforms (Table 2).

Positive percent agreement (PPA) ranged from 0.0% for GenDx (GENEYE, Suzhou City, Jiangsu Province, China) and 24.2% for the immunochromatographic assay (TZcheck) to 80.6% for FlashDetect, which demonstrated the highest overall agreement (κ = 0.822). Other molecular assays showed intermediate performance, with PPA values between 61.8% and 75.0%. Negative percent agreement (NPA) was consistently high across most platforms (≥95%), except for GenDx (80.0%). Overall, agreement ranged from poor (GenDx, κ < 0) to substantial or almost perfect for the best-performing assays. Testing with the GenDx platform was discontinued early because of consistently poor and non-reproducible performance, resulting in a smaller sample size for this assay.

Among culture-positive cases at delivery, seven samples yielded negative results across all molecular assays. Among these seven cases, two women had been classified as GBS-negative at antenatal screening (35–37 weeks), indicating late acquisition of colonisation. Notably, both cases occurred after rupture of membranes lasting 9 and 10 h, respectively. In one of these two cases, mild blood contamination of the swab was also observed. The remaining five cases showed no obvious macroscopic sample abnormality at laboratory inspection.

Visible blood contamination was reported in 22 of 104 samples (21.2%). Among these, the above-mentioned case represented the only instance of a culture-positive sample with negative results across all molecular assays. Other blood-stained samples either yielded concordant results across methods or isolated positive molecular results without culture confirmation. No consistent association was observed between visible blood contamination and systematic false-negative molecular results.

In contrast, one intrapartum sample was culture-negative but yielded positive results across four independent molecular assays. No recent antibiotic exposure, probiotic use, or intravaginal treatment was reported in this patient.

In addition, several intrapartum samples yielded positive results with a single molecular assay without confirmation by other platforms or by culture. These isolated molecular-positive results were predominantly observed with GenDx (*n* = 6) and GenPad (Mirai Genomics Inc., Yokohama-shi, Japan) (*n* = 3), with one case observed with easyNat (Ustar Biotechnologies Ltd., Hangzhou, Zhejiang, China). No consistent clinical or sample-related factors were identified in these cases.

## 4. Discussion

*Streptococcus agalactiae* is a facultative Gram-positive bacterium that forms part of the normal gastrointestinal and genitourinary microbiota. The gastrointestinal tract is considered the primary reservoir of GBS and the source of secondary genital colonisation. Vaginal and rectal carriage may be intermittent, transient, or persistent, with an overall prevalence ranging from 10% to 30% among pregnant women [9,10]. Geographical variation in maternal GBS colonisation has been reported [11]. These differences are particularly relevant in our setting, as the study was conducted in a public secondary-care hospital serving a socioeconomically and ethnically diverse population.

Maternal rectovaginal colonisation at the time of delivery remains the primary risk factor for neonatal early-onset disease due to GBS. The risk of vertical transmission is closely related to the presence and intensity of maternal colonisation during labour [2]. High bacterial load and several intrapartum factors, including prolonged rupture of membranes and prematurity, have been associated with increased risk of neonatal infection [12,13,14,15,16,17,18,19]. As a result, women with documented GBS bacteriuria during pregnancy, considered a marker of high bacterial load, were excluded from the present study.

However, antenatal screening performed at 35–37 weeks of gestation does not necessarily reflect colonisation status at the time of delivery. GBS carriage is dynamic, with acquisition and clearance occurring over relatively short periods. As a result, antenatal screening does not always accurately predict intrapartum colonisation status [20,21,22]. Non-molecular rapid tests, such as immunochromatographic assays, have also demonstrated insufficient sensitivity for reliable intrapartum screening, a finding that was confirmed in our study with the TZCheck assay [23]. Conventional culture-based methods, although considered the reference standard, are not compatible with real-time clinical decision-making. Standard workflows require selective enrichment in LIM broth followed by subculture on solid media such as Granada agar, with plate readings at 24 and 48 h [5]. As a result, the total turnaround time typically ranges from 48 to 72 h, precluding their use for guiding intrapartum antibiotic prophylaxis. Since the early 2000s, PCR-based detection of GBS has been explored, with excellent analytical performance reported by Bergeron et al. and later by Davies et al. [24,25]. However, these early methods relied on laboratory-based workflows and were not suitable for point-of-care use in routine intrapartum settings.

The subsequent introduction of automated, cartridge-based molecular platforms enabled true intrapartum testing. Among these, the Xpert^®^ GBS assay (Cepheid) was one of the first widely implemented point-of-care molecular systems and has been extensively evaluated since the late 2000s, demonstrating high diagnostic performance in several clinical studies [26,27,28]. More recently, additional cartridge-based molecular platforms have been developed, including the Revogene^®^ GBS assay, which has been evaluated in several intrapartum studies and has shown generally favourable diagnostic performance [7,8,29]. However, beyond these systems, the number of validated rapid molecular assays for intrapartum GBS detection remains limited.

Published data on intrapartum molecular detection of GBS remain heterogeneous. In most intrapartum applications, molecular assays are performed directly from vaginal–rectal swabs without prior enrichment in order to preserve rapid turnaround time. In this context, the more modest agreement observed in our study should be interpreted in light of its pragmatic design, with direct testing from rectovaginal swabs collected at admission, no pre-selection of patients, and inclusion of routine delivery-room conditions.

Table S1 highlights the heterogeneity in reported diagnostic performances of molecular assays for GBS detection, largely driven by differences in study design, reference standards, and pre-analytical conditions.

When restricted to direct intrapartum testing without enrichment, sensitivities are generally lower and more variable, typically ranging from approximately 70% to 90%, as illustrated by studies such as d’Otreppe et al. (2023) and Koliwer-Brandl et al. (2023) [8,30]. In contrast, studies using enrichment broth or composite reference standards consistently report higher sensitivities, often exceeding 95%, reflecting more controlled analytical conditions rather than real-world clinical performance. Importantly, several intrapartum studies include high-risk populations (e.g., PROM, PPROM, or preterm labour), in which variability in bacterial load, sampling conditions, and the presence of inhibitors may further affect assay sensitivity. In this context, the highest positive percent agreement observed in our study was 80.6% under pragmatic real-world intrapartum conditions, using a direct molecular approach without enrichment, with deliberate inclusion of PROM cases and exclusion of antibiotic-treated women and those with prior GBS bacteriuria. This finding aligns with the lower-to-mid range of sensitivities reported in pragmatic intrapartum settings, and contrasts with the higher values observed in enriched or laboratory-based evaluations. These results underscore the performance gap between controlled validation studies and real-world labour ward conditions, and support the need for local, pragmatic evaluation when implementing direct molecular POCT strategies for intrapartum GBS screening.

In our evaluation of several commercially available molecular diagnostic platforms for intrapartum GBS detection, we observed substantial variability in performance across assays, including some exhibiting markedly poor and inconsistent results. Notably, one platform (GenDx) showed no detectable positive agreement with culture in our cohort, combined with reduced negative agreement and a negative kappa coefficient, indicating performance below chance level. In addition, results appeared inconsistent and poorly reproducible during the evaluation, leading to early discontinuation of testing with this assay.

Across the remaining molecular platforms, positive agreement ranged from approximately 60% to 80%, despite generally high negative agreement. These findings highlight that assay performance cannot be inferred solely from the underlying amplification technology (e.g., PCR or LAMP), and that factors such as assay design, nucleic acid extraction efficiency, and robustness to inhibitors are likely to play a critical role under real-world intrapartum conditions.

Importantly, the level of published evidence supporting these platforms was highly uneven. While some systems, such as EasyNAT, have been evaluated in other infectious disease contexts, several of the platforms assessed in this study appear to have very limited representation in the peer-reviewed literature, even beyond GBS detection. For some, we were unable to identify any peer-reviewed clinical data at all, whether in intrapartum GBS screening or in other diagnostic applications. This lack of published validation is particularly concerning in a clinical context where test results may directly influence time-critical therapeutic decisions. Taken together, these findings underscore the need for rigorous, independent clinical evaluation before implementation, rather than assuming equivalence between commercially available molecular POCT systems.

Beyond analytical performance, the implementation of molecular POCT in the labour ward raises important operational and organisational considerations. In our study, all evaluated platforms were designed as multi-assay systems capable of supporting a broad range of infectious disease tests. While this versatility represents a potential advantage, it may facilitate implementation by allowing the use of a single platform with a standardised workflow across multiple assays, thereby reducing training requirements and operational complexity.

Substantial differences in operational complexity were observed between platforms. Some systems, such as FlashDetect, easyNat, and iPonatic, are designed as highly integrated “sample-to-answer” platforms with minimal hands-on time, making them more compatible with near-patient testing environments. In contrast, other assays required additional manual steps, increasing hands-on time and potential variability. Certain platforms also involve more complex pre-analytical workflows, which may limit their suitability for use by non-laboratory personnel in routine clinical settings.

In practice, labour ward POCT is often performed by healthcare staff with limited laboratory training. The use of multiple platforms may therefore increase the risk of user-related errors, reduce operational efficiency, and complicate maintenance and quality assurance procedures. From a pragmatic perspective, consolidating testing onto a limited number of robust and user-friendly platforms may improve usability, facilitate staff training, and optimise resource allocation. These considerations highlight that the selection of POCT systems should not rely solely on analytical performance, but also on ease of use and integration into clinical workflows. When integrating analytical performance with practical considerations, several platforms, including FlashDetect, easyNat, Revogene, and iPonatic, demonstrated the most favourable overall profiles in our study. The FlashDetect assay showed the highest agreement and the shortest turnaround time, although it currently requires an external sonication step. In contrast, easyNat and iPonatic involve only minimal manual handling and may be more compatible with near-patient use by non-laboratory personnel. The Revogene system, while demonstrating good analytical performance, requires more delicate manual steps, which may limit its suitability for routine use in delivery ward settings. Beyond these operational aspects, technical factors intrinsic to assay design are also likely to contribute to the observed variability. Among these, the efficiency of bacterial lysis may represent a key determinant, particularly in the context of Gram-positive organisms such as GBS. The thick peptidoglycan-rich cell wall of *S. agalactiae* may limit nucleic acid release, especially in direct testing conditions without enrichment, thereby reducing assay sensitivity. In this regard, mechanical lysis approaches such as sonication may play a critical role in improving DNA extraction efficiency [31,32]. Among currently available systems, the GeneXpert platform incorporates an integrated mechanical lysis step, although it was not evaluated in our study due to cost constraints. Among the platforms evaluated in this study, only the Coyote system incorporated a sonication-based lysis step in the configuration provided for evaluation. It should be noted that alternative configurations including mechanical lysis may exist for other platforms, but were not available in our study setting and were therefore not assessed. However, during the initial phase of our evaluation, the Coyote FlashDetect cartridges provided by the manufacturer were not configured to fully enable this feature, resulting in suboptimal performance. Following the identification of this issue, a subsequent evaluation was conducted using cartridges specifically designed to incorporate effective sonication under the same study protocol. Preliminary results from this ongoing cohort (*n* = 32) suggest a marked improvement in performance, with a positive percent agreement of 90% and a negative percent agreement of 91.7%. Notably, the single apparent false-positive result was confirmed by an independent molecular assay (Revogene), suggesting a potential limitation of culture as the reference standard rather than a true lack of specificity. This observation further challenges the assumption that culture represents an infallible reference standard in this setting. However, because multiple assay characteristics differed across platforms, the specific contribution of lysis efficiency could not be formally isolated in this study. Importantly, current European consensus recommendations emphasise that intrapartum molecular assays should achieve high levels of sensitivity (typically ≥ 90%) to ensure clinical reliability, while also acknowledging the increased risk of false-negative results in the absence of enrichment [33]. Although these findings remain preliminary and require confirmation in a larger cohort, they support the hypothesis that efficient bacterial lysis, particularly through mechanical disruption, may represent a key determinant of diagnostic performance in direct intrapartum molecular testing for GBS.

Overall, our findings highlight the intrinsic challenges of intrapartum molecular detection of GBS. As GBS colonisation reflects a carrier state rather than an active infection, bacterial loads may be low and heterogeneous, which can directly impact the sensitivity of molecular assays, particularly in direct testing conditions without enrichment. In addition, intrapartum sampling, especially in the context of PROM, may introduce biological inhibitors and variability related to anatomical sampling sites, further affecting PCR performance. An important limitation of our study is the use of multiple sequential swabs collected for parallel testing across platforms. Because GBS colonisation may be low-density and heterogeneously distributed, sequential sampling may have introduced inter-swab variability and contributed to discordant results between assays. In addition, sampling was performed by multiple clinicians under routine conditions, which may also have introduced inter-operator variability. These observations indicate that discordance between molecular assays and culture reflects intrinsic limitations of intrapartum GBS testing, rather than an isolated observation specific to our study. Importantly, comparison with enriched culture methods remains problematic, as enrichment enhances bacterial recovery and may artificially inflate the apparent sensitivity gap with direct molecular assays. A similar phenomenon is well described in other areas of microbiology, such as the detection of *Salmonella* in stool samples, where PCR performance may be affected by inhibitors while enrichment-based culture improves sensitivity [34]. Although the GeneXpert platform is often considered a reference in intrapartum GBS molecular testing, it was not included in this study due to local logistical constraints. Importantly, the aim of this work was not to compare assays against a single established system, but to assess the real-world performance of a range of available platforms, including several with limited or no prior clinical evaluation. From a clinical perspective, our results confirm that intrapartum molecular testing should not be interpreted in isolation. A pragmatic diagnostic approach may include: first, consideration of antenatal GBS screening results; second, intrapartum molecular testing at admission when reassessment is clinically indicated; and third, integrated interpretation of results in light of clinical risk factors such as PROM. In this context, positive molecular results may support intrapartum colonisation, whereas negative results should be interpreted with caution, particularly in women with prior GBS positivity or risk factors, as false-negative results may occur. This is supported by our findings, in which several culture-positive samples yielded negative results across all evaluated molecular assays. Conversely, we also identified women who were negative at antenatal screening but culture-positive at delivery, highlighting the dynamic nature of GBS colonisation. Such an approach may lead to overtreatment in some cases, particularly in women who may have cleared colonisation, but it prioritises neonatal safety in a time-critical setting. Looking forward, the development of maternal GBS vaccines may ultimately transform prevention strategies and reduce reliance on intrapartum screening approaches [35]. However, until such strategies become widely available, optimisation and critical evaluation of existing diagnostic tools remain essential.

## 5. Conclusions

This study demonstrates substantial variability in intrapartum GBS detection when assays are used under real-world conditions without prior enrichment, with positive percent agreement ranging from 0.0% to 80.6%. These results indicate that performance cannot be assumed equivalent across platforms, even when based on similar amplification technologies.

Our data further suggest that pre-analytical factors, particularly the efficiency of bacterial lysis, may play a key role in determining sensitivity in direct testing conditions.

In a context where new molecular POCT platforms are rapidly emerging, these observations underline the responsibility of clinical laboratories to independently and rigorously evaluate such systems prior to implementation, as their analytical reliability may vary considerably and may not always meet clinical requirements.

## Figures and Tables

**Table 1:** Technical characteristics of the evaluated assays for intrapartum GBS detection.

Assay	Manufacturer	Method	Reported TAT (min)	Pre-Analytical Processing
TZcheck	Hangzhou	ICT	~15	Manual incubation step
iPonatic	Sansure	PCR	~75	None
FlashDetect	Coyote	PCR	~28	Mechanical lysis (sonication)
easyNat	U-Star	LAMP	~50	None
GenPad	Mirai	PCR	~40	Manual lysis
Revogene	Aidian	PCR	~78	None
GenDx	GENEYE	LAMP	~22	Manual lysis

Abbreviations: ICT—immunochromatographic test; PCR—polymerase chain reaction; LAMP—loop-mediated isothermal amplification; TAT—turnaround time. TAT values are approximate manufacturer-reported turnaround times and may not reflect total workflow duration, including sample handling and pre-analytical steps. Pre-analytical processing refers to manual or mechanical steps required prior to amplification or detection, as specified by the manufacturer.

**Table 2:** Performance of rapid assays relative to conventional intrapartum culture for GBS detection.

Assay	N	PPA % (95% CI)	NPA % (95% CI)	PPV % (95% CI)	NPV % (95% CI)	κ (95% CI)
FlashDetect	102	80.6 (65.0–90.2)	98.5 (91.8–99.9)	96.7 (83.3–99.4)	90.3 (81.3–95.3)	0.822 (0.734–0.910)
easyNat	103	75.0 (59.0–86.3)	95.5 (87.5–98.8)	90.0 (74.4–96.5)	87.7 (78.2–93.3)	0.734 (0.625–0.843)
Revogene	97	73.5 (57.1–85.8)	96.8 (89.2–99.2)	92.6 (76.6–98.0)	87.1 (77.3–93.2)	0.739 (0.628–0.850)
iPonatic	98	69.7 (52.9–82.4)	100.0 (94.5–100.0)	100.0 (85.7–100.0)	86.7 (77.2–92.6)	0.753 (0.648–0.858)
GenPad	100	61.8 (45.0–76.1)	95.5 (87.3–98.8)	87.5 (69.0–95.7)	82.9 (73.0–89.7)	0.616 (0.489–0.743)
TZcheck	96	24.2 (12.8–40.9)	100.0 (94.3–100.0)	100.0 (67.6–100.0)	71.6 (61.4–80.0)	0.296 (0.151–0.441)
GenDx	42	0.0 (0.0–22.8)	80.0 (62.6–90.9)	0.0 (0.0–39.0)	66.7 (49.8–80.4)	−0.235 (−0.452–0.018)

Abbreviations: PPA—positive percent agreement; NPA—negative percent agreement; PPV—positive predictive value; NPV—negative predictive value; κ—Cohen’s kappa coefficient.

## Data Availability

The data presented in this study are not publicly available due to privacy and ethical restrictions, as they contain sensitive patient information. Data may be made available from the corresponding author upon reasonable request and with permission of the relevant ethics committee.

## Supplementary Materials

The following supporting information can be downloaded at: https://www.mdpi.com/article/10.3390/microorganisms14051060/s1, Table S1: Summary of published studies evaluating molecular assays for intrapartum detection of Group B Streptococcus (GBS).
