# Supplementary material for: Intrapartum Molecular Detection of Group B Streptococcus: Real-World Evaluation of Multiple Point-of-Care Platforms and the Potential Role of Lysis Efficiency
Source: Microorganisms. 2026 May 8;14(5):1060. doi: 10.3390/microorganisms14051060 (PMC13209353; doi:10.3390/microorganisms14051060)
Supplement: Supplementary file 1 [file microorganisms-14-01060-s001.zip › microorganisms-4266252-supplementary.pdf]

Supplementary Materials:

Table S1. Summary of published studies evaluating molecular assays for intrapartum detection of Group B Streptococcus (GBS).

Authors / Year	Test / Platform	Sensitivity (%)	Specificity (%)	Index Test Enrichment	Reference Standard	PROM (PPROM) Reporting	Notes / Clinical Context
Gavino & Wang, 2007 **(1)**	Xpert GBS	95.8 (76.9-99.8)	64.5 (45.4-80.2)	No (direct intrapartum)	Antenatal culture	Not specified	Small prospective study (n=55); low specificity likely due to imperfect reference
El Helali et al., 2009 **(2)**	Xpert GBS	98.5	99.6	No (direct intrapartum)	Intrapartum culture	Included (high-risk incl. PROM/preterm)	Large cohort (n≈900); strong intrapartum performance; antenatal PPV low
Bourgeois-Nicolaos et al., 2013 **(3)**	Xpert GBS	~92 (composite)	~98	No (direct; amniotic fluid)	Composite (culture + PCR)	Targeted population (term PROM)	Focus on PROM; different sample type (amniotic fluid)
Helmig & Gertsen, 2017 **(4)**	Xpert GBS	100 (86.3-100)	97.5 (91.3-99.7)	No (direct intrapartum)	Culture (likely enriched)	Included (PROM/PPROM >14h)	High-risk intrapartum population; very low invalid rate
Rabaan et al., 2017 **(5)**	Xpert GBS (modified)	100	89.4	Yes (Todd-Hewitt enrichment)	Enriched culture	Not specified	Modified protocol; not representative of true POCT
Plainvert et al., 2018 **(6)**	Xpert GBS	~78	High	No (direct intrapartum)	Intrapartum culture	Not specified	Women antenatally positive; persistence rate only ~59%
Fullston et al., 2019 **(7)**	Xpert GBS	Not reported	Not reported	No (direct intrapartum)	Not applicable (clinical impact)	Included (pre-labour ROM)	Reduced unnecessary antibiotics (~70%)
Shin & Pride, 2019 **(8)**	Panther Fusion, Aries, Xpert GBS LB	~99-100	High	Yes (enriched LB)	Enriched culture	Not specified	Analytical comparison of NAATs; not POCT setting
Andreasen et al., 2019 **(9)**	BD MAX; GenomEra	84.6 (BD MAX); 71.4 (GenomEra)	High	No (direct intrapartum)	Culture (± enrichment)	Not specified	Intrapartum; GenomEra easier for ward use
Berry et al., 2019 **(10)**	Panther Fusion vs BD MAX	~99% agreement	High	Yes (enriched)	Enriched culture	Not specified	Antenatal screening; high-throughput platforms
Helmig & Gertsen, 2019 **(11)**	Xpert GBS	Not reported	Not reported	No (direct intrapartum)	Not applicable (clinical impact)	Included (PROM >14h)	Antibiotic reduction ~40%
Vieira et al., 2019 **(12)**	Xpert GBS vs qPCR	53 (vs qPCR)	93	Likely direct	qPCR (high-sensitivity reference)	Not specified	High apparent prevalence (51%); sensitivity depends on reference
Choera et al., 2020 **(13)**	Revogene GBS LB vs Xpert GBS LB	PPA 98.0	NPA 96.5	Yes (enriched LB)	Enriched culture	Not specified	High concordance; not direct POCT

Zanini da Rocha et al., 2020 **(14)**	Xpert GBS	86.6	High	Likely direct	Granada culture	Not specified	High-risk cohort; PCR more sensitive than culture
Nielsen et al., 2020 **(15)**	GeneXpert; GenomEra	~91-92	~97-98	Mixed (direct + enriched)	Culture ± enrichment	Included (PROM/PPROM ≥18h)	Intrapartum high-risk; similar performances
Dakin et al., 2022 **(16)**	GeneXpert (survey)	Not reported	Not reported	Variable	Not applicable	Variable	National survey; heterogeneous practices
Thwe et al., 2022 **(17)**	Xpert GBS LB XC	99.3 (composite); 99.1 (culture)	98.7; 91.8	Yes (enriched LB)	Culture + composite	Not specified	New generation assay; controlled conditions
Koliwer-Brandl et al., 2023 **(18)**	Xpert; GenomEra	71.4 (Xpert); 88.1 (GenomEra)	98.6; 99.1	No (direct vaginal)	Culture	Not specified	Direct testing; GenomEra more sensitive but more repeats
d'Otreppe et al., 2023 **(19)**	Revogene GBS DS	85.7 (66.4-95.3)	99.1 (97.3-99.8)	No (direct intrapartum)	Culture (enriched)	Likely included (labour ward setting)	Multicentre (n=398); invalid ~11%
Guetat et al., 2025 **(20)**	GeneXpert	Not reported	Not reported	No (direct intrapartum)	Not applicable (clinical impact)	Included (likely PROM cases)	22.4% incomplete prophylaxis (rapid labour)
Li et al., 2025 **(21)**	CRISPR/Cas13a (PalmCS)	97.5	100	No (direct)	Culture/PCR	Not specified	Emerging portable POCT; small sample (n=40)

Abbreviations: PROM, premature rupture of membranes; PPROM, preterm premature rupture of membranes; PPA, positive percent agreement; NPA, negative percent agreement; NAATs, nucleic acid amplification tests.

References

1. Gavino M, Wang E. A comparison of a new rapid real-time polymerase chain reaction system to traditional culture in determining group B streptococcus colonization. Am J Obstet Gynecol. 2007 Oct;197(4):388.e1-4. doi:10.1016/j.ajog.2007.06.016 PubMed PMID: 17904971.

2. El Helali N, Nguyen JC, Ly A, Giovangrandi Y, Trinquart L. Diagnostic accuracy of a rapid real-time polymerase chain reaction assay for universal intrapartum group B streptococcus screening. Clin Infect Dis Off Publ Infect Dis Soc Am. 2009 Aug 1;49(3):417–23. doi:10.1086/600303 PubMed PMID: 19580414.

3. Bourgeois-Nicolaos N, Cordier AG, Guillet-Caruba C, Casanova F, Benachi A, Doucet-Populaire F. Evaluation of the Cepheid Xpert GBS assay for rapid detection of group B Streptococci in amniotic fluids from pregnant women with premature rupture of membranes. J Clin Microbiol. 2013 Apr;51(4):1305–6. doi:10.1128/JCM.03356-12 PubMed PMID: 23390279; PubMed Central PMCID: PMC3666785.

4. Helmig RB, Gertsen JB. Diagnostic accuracy of polymerase chain reaction for intrapartum detection of group B streptococcus colonization. Acta Obstet Gynecol Scand. 2017 Sep;96(9):1070–4. doi:10.1111/aogs.13169 PubMed PMID: 28504863.

5. Rabaan AA, Saunar JV, Bazzi AM, Soriano JL. Modified use of real-time PCR detection of group B Streptococcus in pregnancy. J Med Microbiol. 2017 Oct;66(10):1516–20. doi:10.1099/jmm.0.000604 PubMed PMID: 28920845.

6. Plainvert C, El Alaoui F, Tazi A, Joubrel C, Anselem O, Ballon M, et al. Intrapartum group B Streptococcus screening in the labor ward by Xpert® GBS real-time PCR. Eur J Clin Microbiol Infect Dis Off Publ Eur Soc Clin Microbiol. 2018 Feb;37(2):265–70. doi:10.1007/s10096-017-3125-2 PubMed PMID: 29082442.

7. Fullston EF, Doyle MJ, Higgins MF, Knowles SJ. Clinical impact of rapid polymerase chain reaction (PCR) test for group B Streptococcus (GBS) in term women with ruptured membranes. Ir J Med Sci. 2019 Nov;188(4):1269–74. doi:10.1007/s11845-019-01977-x PubMed PMID: 30706295.

8. Shin JH, Pride DT. Comparison of Three Nucleic Acid Amplification Tests and Culture for Detection of Group B Streptococcus from Enrichment Broth. J Clin Microbiol. 2019 Jun;57(6):e01958-18. doi:10.1128/JCM.01958-18 PubMed PMID: 30944190; PubMed Central PMCID: PMC6535594.

9. Andreasen T, Kjølseth Møller J, Rohi Khalil M. Comparison of BD MAX GBS and GenomEra GBS assays for rapid intrapartum PCR detection of vaginal carriage of group B streptococci. PloS One. 2019;14(4):e0215314. doi:10.1371/journal.pone.0215314 PubMed PMID: 30990861; PubMed Central PMCID: PMC6467400.

10. Berry GJ, Zhang F, Manji R, Juretschko S. Comparison of the Panther Fusion and BD MAX Group B Streptococcus (GBS) Assays for Detection of GBS in Prenatal Screening Specimens. J Clin Microbiol. 2019 Nov;57(11):e01034-19. doi:10.1128/JCM.01034-19 PubMed PMID: 31462552; PubMed Central PMCID: PMC6812996.

11. Helmig RB, Gertsen JB. Intrapartum PCR-assay for detection of Group B Streptococci (GBS). Eur J Obstet Gynecol Reprod Biol X. 2019 Oct;4:100081. doi:10.1016/j.eurox.2019.100081 PubMed PMID: 31673691; PubMed Central PMCID: PMC6817646.

12. Vieira LL, Perez AV, Machado MM, Kayser ML, Vettori DV, Alegretti AP, et al. Group B Streptococcus detection in pregnant women: comparison of qPCR assay, culture, and the Xpert GBS rapid test. BMC Pregnancy Childbirth. 2019 Dec 30;19(1):532. doi:10.1186/s12884-019-2681-0 PubMed PMID: 31888631; PubMed Central PMCID: PMC6937909.

13. Choera T, Jung-Hynes B, Chen DJ. Comparative study of Revogene GBS LB assay and GeneXpert GBS LB assay for the detection of group B Streptococcus in prenatal screening samples. BMC Infect Dis. 2020 Jan 14;20(1):38. doi:10.1186/s12879-019-4756-y PubMed PMID: 31937247; PubMed Central PMCID: PMC6958782.

14. Zanini da Rocha J, Feltraco J, Radin V, Vitola Gonçalves C, Almeida da Silva PE, Von Groll A. Streptococcus agalactiae colonization and screening approach in high-risk pregnant women in southern Brazil. J Infect Dev Ctries. 2020 Apr 30;14(4):332–40. doi:10.3855/jidc.12025 PubMed PMID: 32379709.

15. Nielsen SY, Møller JK, Khalil MR. A comparison of GenomEra® GBS PCR and GeneXpert ® GBS PCR assays with culture of GBS performed with and without broth pre-enrichment. Eur J Clin Microbiol Infect Dis Off Publ Eur Soc Clin Microbiol. 2020 Oct;39(10):1945–50. doi:10.1007/s10096-020-03934-4 PubMed PMID: 32535806; PubMed Central PMCID: PMC7497322.

16. Dakin A, Ferguson W, Drew R, McCallion N, Higgins MF, Eogan M. Assessing standards for prevention of early onset group B streptococcal (GBS) disease in Ireland. Ir J Med Sci. 2022

Apr;191(2):785–91. doi:10.1007/s11845-021-02639-7 PubMed PMID: 33988805; PubMed Central PMCID: PMC8120250.

17. Thwe PM, Faron ML, Pride DT, Cruz A, Gerstbrein D, Nahmod KA, et al. Multicenter Evaluation of the Cepheid Xpert GBS LB XC Test. J Clin Microbiol. 2022 Dec 21;60(12):e0135622. doi:10.1128/jcm.01356-22 PubMed PMID: 36472424; PubMed Central PMCID: PMC9769564.

18. Koliwer-Brandl H, Nil A, Birri J, Sachs M, Zimmermann R, Zbinden R, et al. Evaluation of two rapid commercial assays for detection of Streptococcus agalactiae from vaginal samples. Acta Obstet Gynecol Scand. 2023 Apr;102(4):450–6. doi:10.1111/aogs.14519 PubMed PMID: 36772902; PubMed Central PMCID: PMC10008276.

19. d'Otreppe S, Lefèvre P, Meex C, Devey A, Sacheli R, Gerard M, et al. Multicenter Performance Evaluation of the Revogene® GBS DS Real-Time PCR Assay for Group B Streptococcus Detection During Labor. Mol Diagn Ther. 2023 Sep;27(5):611–20. doi:10.1007/s40291-023-00660-3 PubMed PMID: 37470972.

20. Guetat C, Roussel L, De Antonio M, Accoceberry M, Houlle C, Petillon F, et al. Does delocalised PCR for Streptococcus B in the labour ward allow adequate administration of antibiotics to prevent early neonatal infection? Braz J Infect Dis Off Publ Braz Soc Infect Dis. 2025;29(4):104553. doi:10.1016/j.bjid.2025.104553 PubMed PMID: 40413980; PubMed Central PMCID: PMC12159896.

21. Li T, Chen S, Chen X, Yang S, Huang H, Xu J, et al. CRISPR-based sensing platform for the Group B streptococcus screening in pregnant women. Anal Chim Acta. 2025 Oct 8;1370:344390. doi:10.1016/j.aca.2025.344390 PubMed PMID: 40750194.
